# Comparative clinical efficacy and safety of the proposed biosimilar ABP 710 with infliximab reference product in patients with rheumatoid arthritis

**DOI:** 10.1186/s13075-020-2142-1

**Published:** 2020-03-26

**Authors:** Mark C. Genovese, Juan Sanchez-Burson, MyungShin Oh, Eva Balazs, Jeffrey Neal, Andrea Everding, Tomas Hala, Rafal Wojciechowski, Gary Fanjiang, Stanley Cohen

**Affiliations:** 1 Division of Immunology and Rheumatology, Stanford University, 1000 Welch RD #203, Palo Alto, CA USA; 2 Hospital Infanta Luisa, Calle San Jacinto 87, Sevilla, Spain; 3 Biosimilars, Amgen, One Amgen Center Dr., Thousand Oaks, CA USA; 4 Csongrád Megyei dr. Bugyi István Kórház Mozgásszervi Rehabilitációs, Sima Ferenc u. 44-58, Csongrad, Hungary; 5 Bluegrass Community Research, 330 Waller Avenue, Lexington, KY USA; 6 Hamburger Rheuma Forschungszentrum II, Hamburg, Germany; 7 CCR Czech a.s., Trida miru 2800, 53002 Pardubice, Czech Republic; 8 Department of Rheumatology and Connective Tissue Diseases, University Hospital No. 2, Bydgoszcz, Poland; 9 Metroplex Clinical Research, 8144 Walnut Hill Lane, Dallas, TX USA

**Keywords:** ABP 710, Infliximab, Biosimilar, Rheumatoid arthritis

## Abstract

**Background:**

ABP 710 is being developed as a biosimilar to infliximab reference product (RP). Analytical similarity and pharmacokinetic equivalence between the two have been previously demonstrated. Here we report results from a comparative clinical study that evaluated the efficacy and safety of ABP 710 relative to the RP in patients with rheumatoid arthritis (RA).

**Methods:**

In this multicenter, randomized, double-blind, 50-week equivalence study, patients with moderate to severe active RA despite methotrexate received 3-mg/kg infusions of ABP 710 or RP at predetermined intervals based on initial randomization and then with re-randomization at week 22. The primary endpoint was response difference (RD) of ACR20 at week 22, with clinical equivalence evaluated based on 90% CI of − 15%, 15%. Secondary endpoints included Disease Activity Score 28-joint count C-reactive protein (DAS28-CRP), ACR20, ACR50, and ACR70 across time, as well as safety and immunogenicity assessments.

**Results:**

A total of 558 patients were randomized for the initial treatment (ABP 710 *n* = 279; RP *n* = 279). The estimated RD of ACR20 at week 22 was 9.37% with 90% CI (2.67%, 15.96%). The lower bound was within the pre-specified criteria, thus confirming non-inferiority; the upper bound exceeded the pre-specified criteria by 0.96% such that superiority could not be ruled out statistically. In a post hoc analysis with adjustment for random imbalance in baseline factors, the CI of RD was narrowed (0.75%, 13.62%). Changes from baseline in DAS28-CRP as well as ACR20, ACR50, and ACR70 response rates across time and hybrid ACR evaluations were similar for the initial and initial/re-randomized treatment groups. Adverse events and incidence of anti-drug antibodies were similar between treatment groups.

**Conclusions:**

These efficacy and safety results support similarity with no clinically meaningful differences between ABP 710 and infliximab RP. Although we were unable to statistically confirm non-superiority, post hoc analysis was supportive of non-superiority. DAS28-CRP, ACR20, ACR50, ACR70, and hybrid ACR evaluations over the entire study were consistently comparable as were safety and immunogenicity.

**Trial registration:**

ClinicalTrials.gov. Identifier: NCT02937701. Registered August 30, 2016.

## Background

ABP 710 is being developed as a biosimilar to infliximab reference product (RP) (Remicade®). Remicade® is approved in the USA and EU for the treatment of adult and pediatric Crohn’s disease, adult and pediatric ulcerative colitis, rheumatoid arthritis (RA), ankylosing spondylitis, psoriatic arthritis, and plaque psoriasis [1, 2]. Biologics have revolutionized the treatment of autoimmune disorders; however, they are expensive options, leading to limited access to treatment. Recently, in an effort to provide alternative treatment options, regulatory agencies have established guidelines to provide an abbreviated development and approval pathway for biosimilars. Guidance from both the US Food and Drug Administration (FDA) and the European Medicines Agency (EMA) outline a stepwise, totality-of-evidence-based approach to the development of a proposed biosimilar, with evaluation of any residual uncertainty about biosimilarity at each step [3–5]. The evaluation of biosimilarity begins with demonstration of analytical (structural, functional, and physiochemical) similarity, which forms the foundation of biosimilarity. This is then followed by comparative preclinical and clinical pharmacology evaluations, including human pharmacokinetics (PK) and pharmacodynamics, if relevant, and finally at least one comparative clinical study to evaluate the similarity of efficacy, safety, and immunogenicity in a representative indication using a sensitive patient population and endpoints to complete the totality of evidence.

ABP 710 and infliximab RP are both chimeric immunoglobulin G monoclonal antibodies produced by recombinant DNA technology, with the same primary amino acid sequence, product strength, dosage form, and formulation upon reconstitution. ABP 710 is similar in secondary and tertiary structure as well as overall conformational stability [6]. The similarity of ABP 710 with infliximab RP for in vitro binding to tumor necrosis factor alpha, FcRn, and FcγRIIIa and in vitro effector function activity of antibody-dependent cell-mediated cytotoxicity and complement-dependent cytotoxicity has been demonstrated through multiple sensitive biological characterization assays (Saleem R, Cantin G, Wikstroem M, Bolton G, Kuhns S, McBride HJ, Liu J: ABP 710: analytical and functional similarity with infliximab reference product, *submitted*). In a clinical PK study in healthy adult patients, similarity of ABP 710 with infliximab RP was demonstrated through comparisons of area under the serum concentration-time curve from time 0 extrapolated to infinity [7]. This comparative clinical study was designed to demonstrate the similarity of ABP 710 with infliximab RP in efficacy, safety, and immunogenicity in patients with moderate to severe RA.

## Methods

### Study design

This was a randomized, double-blind, active-controlled study in adult patients with moderate to severe RA who have an inadequate response to methotrexate (MTX). Approval for this study was granted by the Human Research Ethics Committee and was conducted accordingly. The study protocol was approved by an independent ethics committee or institutional review board at each site prior to study initiation. A total of 558 patients (279 per treatment group) were randomized. Screening occurred ≤ 4 weeks before dosing. Eligible patients were randomized in a ratio of 1:1 to receive 3 mg/kg intravenous (IV) infusion of either ABP 710 or infliximab RP on day 1 (week 0), at weeks 2 and 6, and every 8 weeks thereafter until week 22. At week 22, patients initially randomized to infliximab RP were re-randomized in a 1:1 ratio to either continue receiving infliximab RP every 8 weeks (referred to as RP/RP treatment group) or to transition to receive ABP 710 every 8 weeks (referred to as RP/ABP 710 treatment group) through week 46, while patients initially randomized to ABP 710 continued receiving the same treatment every 8 weeks through week 46 (referred to as ABP 710/ABP 710 treatment group).

### Study population

Eligible patients included infliximab RP- or infliximab biosimilar-naive adult men and women aged 18–80 years with a diagnosis of RA (duration of at least 3 months). Patients were to have active RA, defined as ≥ 6 swollen joints and ≥ 6 tender joints (based on 66/68 joint count excluding distal interphalangeal joints) at screening and baseline and at least one of the following at screening: erythrocyte sedimentation rate ≥ 28 mm/h or serum C-reactive protein (CRP) > 1.0 mg/dl. Patients were to have a positive rheumatoid factor and/or anti-cyclic citrullinated peptide at screening and were to have taken MTX for ≥12 consecutive weeks and be on a stable oral or subcutaneous dose of 7.5 to 25 mg/week MTX for ≥ 8 weeks prior to dosing of investigational product (IP). Other permitted concomitant treatments included continuation of stable dose of oral corticosteroids at a dose of ≤ 10 mg prednisone (or equivalent) per day. Nonbiologic disease-modifying antirheumatic drugs (other than MTX) any biologic treatment for RA as well as other specified treatments that could impact RA were prohibited. Premedications were selected according to local practices and/or the approved product labeling for infliximab and were to be administered approximately 30 min prior to the start of the IV infusion.

### Efficacy endpoints

The primary efficacy endpoint was the response difference (RD) of achieving a 20% improvement from baseline in the American College of Rheumatology core set of measurements (ACR20) at week 22 [8, 9]. Secondary efficacy endpoints included the difference in means of Disease Activity Score 28-joint count-CRP (DAS28-CRP) and the RD for ACR20, ACR50, and ACR70 (20%, 50%, 70% improvement in ACR core set of measurements) at various time points throughout the study.

### Safety

Key safety endpoints included adverse events (AEs), serious adverse events (SAEs), and incidence of antidrug antibodies (ADAs). AEs of interest were determined by customized queries or the Standard Medical Dictionary for Regulatory Activities (MedDRA) queries.

ADAs were assessed at baseline and weeks 2, 6, 14, 22, 30, 34, 38, and 50/end of study. Binding and neutralizing ADAs were detected with a two-tiered approach that included a screening assay and a confirmatory assay. Validated immunoassays were used to detect antibodies capable of binding ABP 710 or infliximab RP. Samples that tested positive for binding ADAs were subsequently tested in the corresponding target binding assay to determine neutralizing activity against ABP 710 or RP.

### Statistical analyses

A sample size of approximately 550 patients was chosen to achieve > 90% power to demonstrate equivalence between the ABP 710 and infliximab RP groups for the primary efficacy endpoint, RD of ACR20 at week 22, with a two-sided significance level of 0.05 and equivalence margin of (− 15%, 15%). This calculation was based on the assumption of an expected ACR20 response of 52% at week 22 for each group.

All efficacy endpoints were analyzed using the intent-to-treat (ITT) analysis set, which included all randomized patients, based on patients’ randomized treatment, regardless of the actual treatment received. The per-protocol (PP) analysis set included all patients randomized in the study who completed the treatment period and did not experience a protocol deviation that affected their evaluation for the primary objective of the study and was based on actual treatment received. The analysis of safety endpoints included all randomized patients who received any amount of IP and was based on actual treatment received (safety analysis set).

Clinical equivalence for the primary endpoint was evaluated by comparing the two-sided 90% confidence interval (CI) for RD of ACR20 at week 22 between ABP 710 and infliximab RP with an equivalence margin of (− 15%, 15%). RD was estimated by the Mantel-Haenszel estimate and the CIs for RD of ACR20 were estimated by the stratified Newcombe confidence limits adjusting for two stratification factors (geographic region and prior biologic use for RA) on the ITT analysis set with non-responder imputation (NRI).

An additional sensitivity analysis was performed ad hoc to adjust for the impact of a random imbalance in baseline demographic and disease characteristics between the 2 groups [10–12]. The baseline covariates identified to be predictive of ACR20 by a stratified conditional logistic regression (with forward selection method and *p* value ≤ 0.25 to enter) were identified for each analysis performed in the ITT set (with NRI, last observation carried forward, and as observed) and the PP set.

For the secondary endpoints of RD of ACR20 (at scheduled visits other than week 22), RD of ACR50, and RD of ACR70, analyses were performed on the ITT analysis set with NRI using the same statistical model as used for the primary analysis of the primary endpoint. For DAS28-CRP change from baseline, the difference between means and the corresponding CIs are estimated using an ANCOVA model adjusted for the baseline DAS28-CRP and the two stratification factors on ITT analysis set with observed data.

## Results

### Patient disposition

**Fig. 1:**
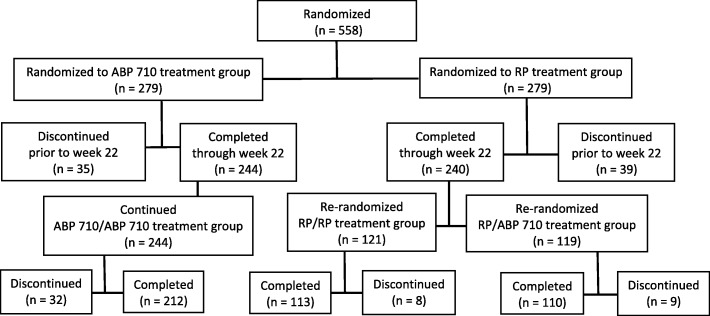
Patient disposition

Subject disposition is summarized in Fig. 1. A total of 558 patients (279 in the ABP 710 treatment group and 279 in the infliximab RP treatment group) were randomized at 75 centers across 9 countries. Overall, 484 out of 558 (86.7%) patients completed the study through week 22 and were re-randomized; 74 (13.3%) patients discontinued the study prior to week 22 and were not re-randomized. For both treatment groups, the most common reason for discontinuing the study prior to week 22 was due to AEs (11 [3.9%] patients in the ABP 710 treatment group and 14 [5.0%] patients in the infliximab RP treatment group). Of the 484 patients who were re-randomized at week 22, 244 were initially randomized to ABP 710 and, thus, continued to receive ABP 710 (ABP 710/ABP 710 treatment group); 121 were initially randomized to infliximab RP and were re-randomized to continue receiving infliximab RP (RP/RP treatment group); and 119 initially randomized to infliximab RP were re-randomized to receive ABP 710 (RP/ABP 710 treatment group). Overall, 435 (78.0%) of the 558 patients who were initially randomized completed the study, and 123 (22.0%) patients discontinued the study. For both treatment groups, the most common reason for discontinuing the study was due to AEs (21 [7.5%] patients in the ABP 710 treatment group and 20 [7.2%] patients in the RP treatment group). The ITT analysis set included 558 patients, the PP analysis set included 471 patients, and the safety analysis set included 556 patients.

### Baseline demographics and clinical characteristics

The majority of patients were female (78.3%) and white (95.3%), with a mean age of 54.9 years (range 19–77 years) and a mean of 8.53 years (range 0.3–45.0 years) since diagnosis (Table 1). Overall, baseline demographics and clinical characteristics were similar across treatment groups with a mean (standard deviation [SD]) baseline DAS28-CRP score of 5.58 (0.912) for ABP 710 and 5.60 (0.893) for infliximab RP. Clinical characteristics at week 22 for the 484 re-randomized patients were also similar across treatment groups with a mean (SD) DAS-CRP score of 5.54 (0.890) for ABP 710/ABP 710, 5.59 (0.900) for RP/RP, and 5.54 (0.913) for RP/ABP 710.

**Table 1:** Summary of demographic data and baseline characteristics

Parameter	ABP 710 (***n*** = 279)	Infliximab RP (***n*** = 279)	Total (***n*** = 558)
Mean age, years (range)	55.0 (23, 77)	54.8 (19, 77)	54.9 (19, 77)
Women, *n* (%)	214 (76.7)	223 (79.9)	437 (78.3)
Ethnicity, *n* (%)			
Hispanic or Latino	18 (6.5)	13 (4.7)	31 (5.6)
Not Hispanic or Latino	261 (93.5)	266 (95.3)	527 (94.4)
Race, *n* (%)			
White	265 (95.0)	267 (95.7)	532 (95.3)
Black or African American	12 (4.3)	12 (4.3)	24 (4.3)
Asian	2 (0.7)	0 (0.0)	2 (0.4)
Region, *n* (%)			
Asia Pacific	5 (1.8)	4 (1.4)	9 (1.6)
Europe	220 (78.9)	222 (79.6)	442 (79.2)
North America	54 (19.4)	53 (19.0)	107 (19.2)
Mean duration of RA, years (range)	8.72 (0.3, 45.0)	8.34 (0.3, 39.0)	8.53 (0.3, 45.0)
Duration of RA category, *n* (%)			
< 5 years	104 (37.3)	123 (44.1)	227 (40.7)
≥ 5 years	175 (62.7)	156 (55.9)	331 (59.3)
Swollen joint count, mean (SD)	14.6 (8.05)	14.7 (8.83)	14.7 (8.44)
Tender joint count, mean (SD)	23.1 (12.16)	23.8 (13.38)	23.4 (12.78)
Subject Global Health Assessment, mean (SD)	65.4 (18.13)	64.1 (20.03)	64.7 (19.10)
Investigator Global Health Assessment, mean (SD)	64.5 (15.88)	64.1 (15.76)	64.3 (15.81)
HAQ-DI, mean (SD)	1.44 (0.584)	1.42 (0.617)	1.43 (0.601)
Serum CRP, mean (SD), mg/L	14.26 (20.171)	14.64 (23.117)	14.45 (21.675)
DAS28-CRP, mean (SD)	5.58 (0.912)	5.60 (0.893)	5.59 (0.902)
RF status, *n* (%)			
Positive at screening	244 (87.5)	251 (90.0)	495 (88.7)
Anti-CCP status, *n* (%)			
Positive at screening	253 (90.7)	253 (90.7)	506 (90.7)
Prior biologic use for RA, *n* (%)	77 (27.6)	81 (29.0)	158 (28.3)
MTX dose, mean (SD), mg/week	17.54 (4.835)	17.19 (4.938)	17.36 (4.886)

*Abbreviations: CCP* cyclic citrullinated peptide, *CRP* C-reactive protein, *DAS28* Disease Activity Score 28-joint count, *HAQ-DI* Health Assessment Questionnaire Disability Index, *MTX* methotrexate, *RA* rheumatoid arthritis, *RF* rheumatoid factor, *RP* reference product, *SD* standard deviation

### Concomitant and previous medications

Prior use of biologics for RA and baseline RA medications were balanced across groups; the majority of patients (ABP 710, 72.4%; infliximab RP, 71.0%) were treatment-naive for prior use of biologics for RA. Oral corticosteroids were used at baseline by 55.6% and 52.0% of patients in the ABP 710 and infliximab RP groups, respectively. Percentages of corticosteroid use at baseline were also similar among re-randomized groups with 54.9%, 52.1%, and 51.3% of ABP 710/ABP 710, RP/RP, and RP/ABP 710 treatment groups, respectively. Baseline mean MTX doses were similar across treatment groups with 17.54 (4.835) mg/week and 17.19 (4.938) mg/week for ABP 710 and infliximab RP, respectively, and 17.51 (4.951), 17.02 (4.703), and 17.50 (5.218) mg/week for ABP 710/ABP 710, RP/RP and RP/ABP 710, respectively.

### Clinical efficacy

#### ACR20 at week 22

At week 22, 68.1% (190/279) of patients in the ABP 710 group and 59.1% (165/279) of patients in the infliximab RP group met the ACR20 response criteria. The estimated RD of ACR20 was 9.37% with a 2-sided 90% CI of 2.67%, 15.96%. The 90% CI exceeded the upper bound of the prespecified equivalence margin (− 15%, 15%). Post hoc analyses of ACR20 at week 22 were used to adjust for the impact of random imbalance in baseline demographic and disease characteristic between the two treatment arms (Table 2). The resulting estimate of RD was reduced to 7.18%, and the 90% CI was narrowed to 0.75%, 13.62%, within the margin of − 15%, 15%.

**Table 2:** Summary of analyses of ACR20 at week 22 using non-parametric analysis of covariance method

Analysis population	RD (ABP 710 – RP) of ACR20 (%)		
	Point estimate	90% CI	95% CI
ITT analysis set with NRI	7.18	0.75, 13.62	− 0.49, 14.85
ITT analysis set with LOCF imputation	6.09	− 0.26, 12.44	− 1.48, 13.66
ITT analysis set, as observed	6.52	0.04, 12.99	− 1.20, 14.23
Per protocol analysis set	8.40	1.73, 15.06	0.45, 16.34

*Abbreviations: ACR20* 20% improvement in American College of Rheumatology core set measurements, *CI* confidence interval, *ITT* intent-to-treat, *LOCF* last observation carried forward, *NRI* nonresponder imputation, *RD* response difference, *RP* reference product

Note: The Mantel-Haenszel estimate and corresponding CIs for the RD were estimated by the non-parametric analysis of covariance method using the SAS NParCov4 macro with strata determined by IXRS values of the stratification factors geographic region (Europe, North America combined with Asia Pacific) and prior biologic use and adjustment for baseline covariates (tender joint count, swollen joint count, Subject’s Global Health Assessment, Investigator’s Global Health Assessment, Subject’s Assessment of Disease-related Pain, HAQ-DI, C-reactive protein, age, use of oral corticosteroid, use of non-steroidal anti-inflammatory drugs, body mass index categories of < 25, 25–30, and ≥ 30, and methotrexate dose)

#### ACR20, ACR50, and ACR70 across time

**Fig. 2:**
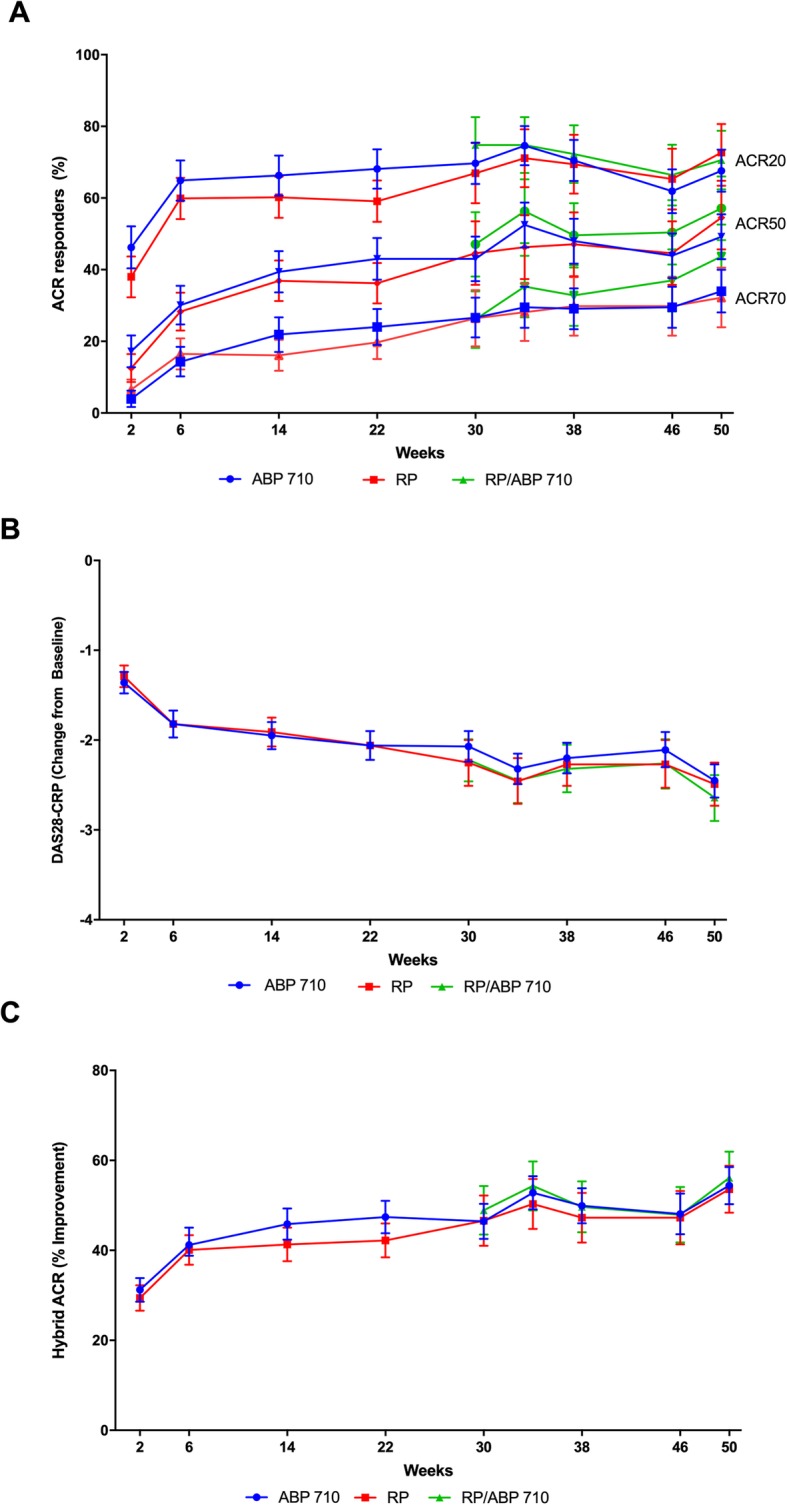
**a** Percentage of patients achieving ACR20, ACR50, and ACR70 by study week, ± 95% CI. ACR20, ACR50, and ACR70 is the 20, 50, and 70%, respectively, improvement from baseline in American College of Rheumatology core set measurements. **b** Mean ± 95% CI change from baseline DAS28-CRP by study week. **c** Percentage of Hybrid ACR improvement from baseline ± 95% CI. Hybrid ACR was calculated when all seven ACR components were non-missing. For post week 22, only re-randomized subjects were included

ACR20, ACR50, and ACR70 response rates with 95% CIs by treatment group through week 22 and post week 22 are summarized in Fig. 2a. Over the entire study, the maximum RD of ACR20 occurred at the week 22 time point, with narrow differences before and directly after this time point. Results show that efficacy was consistently maintained throughout the study and that a single transition from infliximab RP to ABP 710 did not impact efficacy.

#### DAS28-CRP

At week 22, the mean change from baseline in DAS28-CRP was − 2.06 for both groups, with a difference between treatment groups (two-sided 90% CI) of − 0.01 (− 0.20%, 0.17%), further substantiating clinical efficacy equivalence between ABP 710 and infliximab RP. Mean change from baseline in DAS28-CRP decreased similarly throughout the study in both groups, indicating similar reduced disease activity (Fig. 2b). For reference, European League Against Rheumatism (EULAR) previously defined a change in DAS28 of 0.6 as the minimal clinically important difference [13]; the differences between treatment groups were well within this margin throughout the study. In addition, the single transition from infliximab RP to ABP 710 did not impact efficacy.

#### Hybrid ACR

Results for analysis of hybrid ACR through the entire study by treatment groups are shown in Fig. 2c. Hybrid ACR by treatment groups followed the same pattern of response throughout the study with overlapping 95% CIs between the treatment groups at all time points. Results show that efficacy was consistently maintained throughout the study. In addition, the single transition from infliximab RP to ABP 710 did not impact efficacy.

### Safety

Through week 22, 50.7% of all patients had ≥ 1 treatment-emergent AE (TEAE) during the study and the percentages of patients who reported TEAEs were similar among patients in the ABP 710 and RP groups (51.8% and 49.6%, respectively) (Table 3). Post week 22, 55.7% of all patients had ≥ 1 TEAE during the study and the percentages of patients who reported TEAEs were similar among patients in the ABP 710/ABP 710, RP/RP, and RP/ABP 710 groups (53.9%, 57.0%, and 58.0%, respectively) (Table 4).

**Table 3:** Treatment-emergent adverse events (TEAEs) through week 22

	ABP 710 (***N*** = 278) ***n*** (%)	RP (***N*** = 278) ***n*** (%)
Any AE	144 (51.8)	138 (49.6)
Any grade ≥ 3 AE	12 (4.3)	14 (5.0)
Any TEAE	54 (19.4)	58 (20.9)
Any AE with outcome of death	1 (0.4)	1 (0.4)
Any SAE	9 (3.2)	14 (5.0)
Any AE leading to infusion delay/not administered	30 (10.8)	30 (10.8)
Any TEAE leading to discontinuation of IP	16 (5.8)	18 (6.5)
**Adverse events of interest**		
Any AE of interest	39 (14.0)	49 (17.6)
Infusion reactions including hypersensitivity	22 (7.9)	37 (13.3)
Hematological reaction	11 (4.0)	5 (1.8)
Hepatotoxicity	9 (3.2)	9 (3.2)
Serious infections	2 (0.7)	4 (1.4)
Malignancies	2 (0.7)	2 (0.7)
Congestive heart failure	1 (0.4)	0 (0.0)
Opportunistic infections	1 (0.4)	2 (0.7)
Demyelinating disorders	0 (0.0)	0 (0.0)
Hepatitis B reactivation	0 (0.0)	0 (0.0)
Autoimmunity (systemic lupus erythematosus and sarcoid)	0 (0.0)	1 (0.4)
**TEAEs reported in ≥ 5% of patients in any treatment group,*n*(%)**		
Upper respiratory tract infection	17 (6.1)	18 (6.5)
Rheumatoid arthritis	14 (5.0)	11 (4.0)
Nasopharyngitis	12 (4.3)	4 (1.4)
Bronchitis	9 (3.2)	4 (1.4)
Pharyngitis	8 (2.9)	3 (1.1)

*Abbreviations: AE* adverse event, *IP* investigational product, *RP* reference product, *SAE* serious adverse event, *TEAE* treatment-related adverse event

**Table 4:** TEAEs post week 22

	ABP 710/ABP 710 (***N*** = 241) ***n*** (%)	RP/RP (***N*** = 121) ***n*** (%)	RP/ABP 710 (***N*** = 119) ***n*** (%)
Any AE	130 (53.9)	69 (57.0)	69 (58.0)
Any grade ≥ 3 AE	18 (7.5)	7 (5.8)	5 (4.2)
Any TEAE	48 (19.9)	29 (24.0)	26 (21.8)
Any AE with outcome of death	0 (0.0)	0 (0.0)	0 (0.0)
Any SAE	15 (6.2)	4 (3.3)	1 (0.8)
Any AE leading to infusion delay/not administered	19 (7.9)	11 (9.1)	8 (6.7)
Any TEAE leading to discontinuation of IP	12 (5.0)	4 (3.3)	4 (3.4)
**Adverse events of interest**			
Any AE of interest	43 (17.8)	19 (15.7)	16 (13.4)
Infusion reactions including hypersensitivity	20 (8.3)	12 (9.9)	7 (5.9)
Hepatotoxicity	11 (4.6)	4 (3.3)	0 (0.0)
Hematological reactions	10 (4.1)	6 (5.0)	8 (6.7)
Serious infections	5 (2.1)	1 (0.8)	3 (2.5)
Malignancies	2 (0.8)	0 (0.0)	0 (0.0)
Congestive heart failure	1 (0.4)	1 (0.8)	0 (0.0)
Opportunistic infections	0 (0.0)	1 (0.8)	0 (0.0)
Demyelinating disorders	0 (0.0)	0 (0.0)	0 (0.0)
Hepatitis B reactivation	0 (0.0)	0 (0.0)	0 (0.0)
Autoimmunity (systemic lupus erythematosus and sarcoid)	0 (0.0)	0 (0.0)	0 (0.0)
**TEAEs reported in ≥ 5% of patients in any treatment group,*n*(%)**			
Upper respiratory tract infection	23 (9.5)	9 (7.4)	14 (11.8)
Rheumatoid arthritis	23 (9.5)	9 (7.4)	7 (5.9)
Nasopharyngitis	13 (5.4)	11 (9.1)	8 (6.7)
Bronchitis	8 (3.3)	6 (5.0)	2 (1.7)
Pharyngitis	2 (0.8)	2 (1.7)	7 (5.9)

*Abbreviations: AE* adverse event, *RP* reference product, *SAE* serious adverse event, *TEAE* treatment-emergent adverse event

TEAEs reported in ≥ 5% of patients in any treatment group were reported at similar rates in the three treatment groups (ABP 710/ABP 710; RP/RP; RP/ABP 710). In general, the subject incidence rates for these frequently reported AEs were similar across the treatment groups, and no safety signal was associated with a single transition from infliximab RP to ABP 710. The only event with at least a 5% difference between treatment groups was pharyngitis, which was reported in 2 (0.8%), 2 (1.7%), and 7 (5.9%) patients in the ABP 710/ABP 710, RP/RP, and RP/ABP 710 treatment groups, respectively.

For the three treatment groups, the highest subject incidence rates of AEs were in the System Organ Class of infections and infestations. Discontinuation of IP or study due to ≥ 1 AE in the ABP 710/ABP 710, RP/RP, and RP/ABP 710 treatment groups occurred in 12 (5.0%), 4 (3.3%), and 4 (3.4%) patients, respectively. A total of 30 (6.2%) patients reported having AEs of grade ≥ 3 post week 22 and the percentages of patients were similar between the ABP 710/ABP 710 (*n* = 18; 7.5%), RP/RP (*n* = 7; 5.8%), and RP/ABP 710 (*n* = 5; 4.2%) groups. No fatal AEs were reported post week 22. Overall, there were no differences between groups that were ≥ 5% for the percentage of patients who experienced a TEAE.

### Immunogenicity

Through week 22, 556 patients had ≥ 1 evaluable ADA result and were included in the antibody analysis. For the ABP 710 and infliximab RP groups, 16 (5.8%) and 11 (4.0%) patients, respectively, tested positive for pre-existing binding antibodies and no patients tested positive for pre-existing neutralizing antibodies. The number of patients with a binding negative or no result at baseline who tested positive for binding antibodies at week 22 post baseline was similar in each group (ABP 710, *n* = 149 [57.1%]; RP, *n* = 160 [60.0%]). A total of 102 (18.3%) patients tested positive for neutralizing ADAs by week 22, which was also similar to that for each treatment group (ABP 710, *n* = 47 [18.0%]; RP, *n* = 55 [20.8%]). A total of 186 (38.7%) of patients who were re-randomized (96 [39.8%] in the ABP 710/ABP 710 treatment group, 45 [37.2%] in the RP/RP treatment group, and 45 [37.8%] in the RP/ABP 710 treatment group) were negative for binding ADAs at week 22, had a negative or no result before week 22, and had a post week 22 ADA result by week 50. Of these, 66 (35.5%) patients (29 [30.2%] in the ABP 710/ABP 170 treatment group, 19 [42.2%] in the RP/RP treatment group, and 18 [40.0%] in the RP/ABP 710 treatment group) tested positive for binding ADAs post week 22. Six patients (3 [3.1%] patients in the ABP 710/ABP 710 treatment group, 1 [2.2%] subject in the RP/RP treatment group, and 2 [4.4%] patients in the RP/ABP 710 treatment group) tested positive for neutralizing ADAs post week 22. Descriptive results for ADAs by visit and treatment indicate that the incidence of ADAs was similar in patients across all groups throughout the course of the study (Table 5).

**Table 5:** Immunogenicity—total incidence of ADAs

	Antibody positive by week 22 with a negative or no result at baseline		Antibody positive post week 22 with a negative or no result at week 22		
	ABP 710	RP	ABP 710/ABP 710	RP/RP	RP/ABP 710
Number of patients	261	264	96	45	45
Binding antibody, *n* (%)	149 (57.1)	160 (60.6)	29 (30.2)	19 (42.2)	18 (40.0)
Neutralizing antibody, *n* (%)	47 (18.0)	55 (20.8)	3 (3.1)	1 (2.2)	2 (4.4)

*Abbreviations: ADA* antidrug antibody, *RP* reference product

### Pharmacokinetic results

Of the 558 randomized patients, 556 had at least one evaluable result for serum concentration of ABP 710 or infliximab RP at any visit and were included in the PK analysis. PK results revealed that trough serum concentrations were similar between groups across all study weeks, indicating that exposure was similar between treatment groups. The geometric mean trough serum concentrations and geometric coefficient of variation at week 22 were 1126.98 ng/ml (291.55%) and 1184.40 ng/ml (327.94%) for ABP 710 and infliximab RP, respectively, with similar concentrations across groups at all time points up to week 22 with geometric mean ratio values ranging from 0.91 to 1.05, indicating that ABP 710 and infliximab RP have similar PK following multiple-dose administration in patients with RA. The geometric mean trough serum concentrations with geometric coefficient of variation post transition at week 50 were 4028.52 ng/ml (245.05%; ABP 710/ABP 710), 3432.72 ng/ml (437.30%; RP/RP), and 2948.96 ng/ml (587.56%; RP/ABP 710) with similar concentrations across groups at all time points with geometric mean ratio values ranging from 0.90 to 1.25 for comparisons of ABP 710/ABP 710 and RP/RP treatment groups and from 0.72 to 1.15 for comparisons of RP/ABP 710 and RP/RP treatment groups.

## Discussion

In this study, we have established similarity of efficacy of ABP 710 and infliximab RP in patients with moderate to severe RA by demonstrating no clinically meaningful differences between the proposed biosimilar ABP 710 and the infliximab RP. This study design met FDA and EMA guidelines contributing to the development and approval of biosimilar agents. The primary efficacy endpoint was RD of ACR20 at week 22. The 2-sided 90% CI of the RD of ACR20 between ABP 710 and infliximab RP was within the lower bound of the prespecified equivalence margin but exceeded the upper bound by 0.96%. An additional analysis was performed to account for an imbalance in baseline demographic and disease characteristics, which may have contributed to the observed difference between the ABP 710 and RP treatment groups. When covariates identified to be predictive of ACR20 by a stratified conditional logistic regression were selected for adjustment, the resulting estimates of RD of ACR20 at week 22 and the associated 90% CIs were narrowed such that it was contained within the prespecified equivalence margin.

ACR20 is a dichotomous measure used as the primary efficacy endpoint for this study based on consistency with the originator and precedence from prior studies evaluating biosimilars. However, continuous endpoints such as DAS28-CRP and hybrid ACR may be more sensitive for a comparison of two active and very similar products as is the case during clinical evaluation of a proposed biosimilar with its RP. Based on results obtained at week 22 and over the entire study, both DAS28-CRP and hybrid ACR showed minimal differences between treatment groups and are supportive of similarity of ABP 710 and RP. Specifically, at week 22, the difference between treatment groups of ABP 710 and infliximab RP in the DAS28-CRP mean change from baseline was − 0.01 with a 90% CI of − 0.20, 0.17 and a 95% CI of − 0.24, 0.21. This difference of less than 0.6 is consistent with no clinically meaningful difference and aligns with EULAR definition, which states that a change in DAS28-CRP of up to 0.6 points within an individual constitutes no improvement [13].

Further evaluations of efficacy were also consistent with similarity of ABP 710 and RP. When analyzed across time, the maximum RD of ACR20 occurred at the week 22 time point and was the only time point where the upper bound of the prespecified equivalence margin was exceeded. RD CIs were narrow at time points before and after. At weeks 14 and 30, which are also frequent pre-determined time points of primary analysis in RA efficacy studies, the RD of ACR20 is fully contained within the prespecified equivalence margin. These results of RD of ACR20 across time points are consistent with both non-inferiority and non-superiority. In addition, overlapping 95% CIs for RD of ACR50 and ACR70 at all points across time further demonstrate similarity in efficacy. Considering that, for biosimilars, the purpose of clinical evaluation is to confirm similarity with the RP (and not demonstrate efficacy per se), the inconsistent observation at week 22 may not be considered clinically meaningful.

In addition to similarity of efficacy, we have demonstrated further safety and PK similarity for the comparison of ABP 710 and RP. As was consistent with previous results in healthy patients, serum concentrations as well as the subject incidence rates of developing ADAs of ABP 710 and RP were similar between the two treatment groups or the three re-randomized treatment groups throughout the entire study. The frequency, type, and severity of AEs were similar between treatment groups with no clinically meaningful differences. Safety events were consistent with the known safety profile of infliximab with no new or unexpected safety signals and no impact of a single transition from RP to ABP 710.

Several biosimilars of infliximab have been approved in various countries (Remsima™, Celltrion, South Korea; Inflectra™, Pfizer, USA; Infimab, Reliance Life Sciences, India; Renflexis®, Samsung Bioepis Co. Ltd./Merck Sharp & Dohme Corp., USA; Flixabi®, Biogen, USA) [14–16]. As an example, efficacy in RA for CT-P13 (Remsima™, Inflectra™) was also demonstrated in a comparative efficacy study in patients with active disease despite MTX as evaluated by ACR20 but with the primary endpoint evaluated at week 30. In an extension of the study up to week 54 that included efficacy, radiographic progression, immunogenicity, safety, and PK, the conclusion of similarity to infliximab RP was unchanged [17, 18].

Although this study successfully demonstrated that there were no clinically meaningful differences between ABP 710 and the RP with respect to efficacy, exceeding the upper bound margin for the demonstration of efficacy may be considered a limitation. The upper bound margin for the primary endpoint RD of ACR20 at week 22 was 15.96%, which exceeded the prespecified upper limit of 15% by 0.96%. Therefore, while it may be questioned whether this study met its objective, it is important to understand that biosimilarity is based on the totality of evidence that also includes the demonstration of similarity in analytical and preclinical assessments and clinical pharmacology, safety, and immunogenicity. ABP 710 has been successfully shown to be similar to the RP for all of these aspects.

## Conclusions

In conclusion, this randomized, double-blind, active-controlled, multiple-dose, comparative clinical study showed that there were no clinically meaningful differences between ABP 710 and infliximab RP. In addition, this study demonstrated that the safety and immunogenicity of ABP 710 were similar to those of the RP and that efficacy and safety were not impacted by a single switch from infliximab RP to ABP 710. These results, along with the totality of evidence, which includes demonstration of similarity of ABP 710 with the infliximab RP in analytical assessments and PK and safety evaluation in healthy patients, confirm that ABP 710 is similar to infliximab RP.

## Data Availability

There is a plan to share data. This may include de-identified individual patient data for variables necessary to address the specific research question in an approved data-sharing request; also related data dictionaries, study protocol, statistical analysis plan, informed consent form, and/or clinical study report. Data sharing requests relating to data in this manuscript will be considered after the publication date and (1) this product and indication (or other new use) have been granted marketing authorization in both the US and Europe or (2) clinical development discontinues and the data will not be submitted to regulatory authorities. There is no end date for eligibility to submit a data sharing request for these data. Qualified researchers may submit a request containing the research objectives, the Amgen product(s) and Amgen study/studies in scope, endpoints/outcomes of interest, statistical analysis plan, data requirements, publication plan, and qualifications of the researcher(s). In general, Amgen does not grant external requests for individual patient data for the purpose of re-evaluating safety and efficacy issues already addressed in the product labeling. A committee of internal advisors reviews requests. If not approved, a Data Sharing Independent Review Panel may arbitrate and make the final decision. Requests that pose a potential conflict of interest or an actual or potential competitive risk may be declined at Amgen’s sole discretion and without further arbitration. Upon approval, information necessary to address the research question will be provided under the terms of a data sharing agreement. This may include anonymized individual patient data and/or available supporting documents, containing fragments of analysis code where provided in analysis specifications. Further details are available at the following: http://www.amgen.com/datasharing.

## Abbreviations

ADA: Antidrug antibody
AE: Adverse event
CI: Confidence interval
CRP: C-reactive protein
DAS28: Disease Activity Score 28-joint count
IP: Investigational product
ITT: Intent-to-treat
IV: Intravenous
MTX: Methotrexate
NRI: Nonresponder imputation
PK: Pharmacokinetic
PP: Per protocol
RA: Rheumatoid arthritis
RD: Response difference
RP: Reference product
SAE: Serious adverse event
SD: Standard deviation
